# Characterization of a New Heat Dissipation Matric Potential Sensor

**DOI:** 10.3390/s130101137

**Published:** 2013-01-17

**Authors:** Luzius Matile, Roman Berger, Daniel Wächter, Rolf Krebs

**Affiliations:** Institute of Natural Resources Sciences, Zurich University of Applied Sciences, CH-8820 Wädenswil, Switzerland; E-Mails: roman.berger@zhaw.ch (R.B.); daniel.waechter@art.admin.ch (D.W.); rolf.krebs@zhaw.ch (R.K.)

**Keywords:** soil moisture, matric potential, sensor, heat dissipation, temperature, irrigation control

## Abstract

Soil moisture sensors can help to reduce the amount of water needed for irrigation. In this paper we describe the PlantCare soil moisture sensor as a new type of heat dissipation sensor, its calibration and the correction for temperature changes. With the PlantCare sensor it is possible to measure the matric potential indirectly to monitor or control irrigation. This sensor is based on thermal properties of a synthetic felt. After a defined heating phase the cooling time to a threshold temperature is a function of the water content in the synthetic felt. The water content in this porous matrix is controlled by the matric potential in the surrounding soil. Calibration measurements have shown that the sensor is most sensitive to −400 hPa and allows lower sensitivity measurements to −800 hPa. The disturbing effect of the temperature change during the measurement on the cooling time can be corrected by a linear function and the differences among sensors are minimized by a two point calibration.

## 1. Introduction

In many regions of the world water is scarce and the sustainable use of the water resources is indispensable. Almost 40% of the world's food is produced by irrigated agriculture, accounting for 70% of worldwide fresh water use [1]. Water saving irrigation systems play therefore a key role in the sustainable use of water resources today and even more in future. The measurement of the soil moisture is of mayor importance for the controlling of irrigation systems. Although there are numerous soil moisture sensors available there is need for low maintenance and inexpensive matric potential sensors [2].

The matric potential can be measured directly by tensiometers [3] or indirectly by matric potential sensors that measure the water content of a porous matrix that is in equilibrium with the surrounding soil [4]. The measurement of the water content of the porous matrix is either based on the electrical resistance, the dielectric constant (measured either by frequency domain or by time domain reflectometry) or on the heat conductivity. The indirect measurement of the matric potential has the following advantages: almost no maintenance and not so much limited at low matric potentials as tensiometers [5].

Heat dissipation sensors first described by Phene [6] and evaluated by Reece [7] detect the matrix potential indirectly by measuring the water content of a porous media based on its thermal properties. The temperature dependence of the heat conductivity makes the heat dissipation sensors very sensitive to the ambient temperature although corrections of the effect have been developed [8]. The purpose of this paper is the description of the PlantCare sensor as a new heat dissipation sensor, its calibration and the correction for temperature changes.

## 2. Experimental Section

### 2.1. Sensing Principle of the PlantCare Sensor

**Figure 1.**
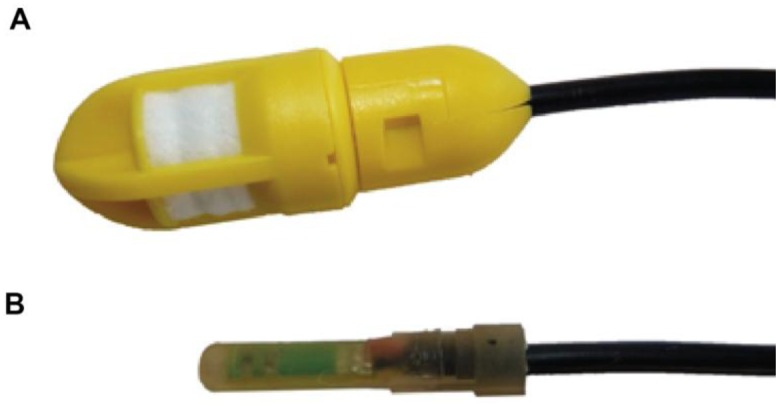
(**A**) PlantCare sensor (diameter 15 mm). (**B**) PlantCare sensor without box and synthetic felt.

**Figure 2.**
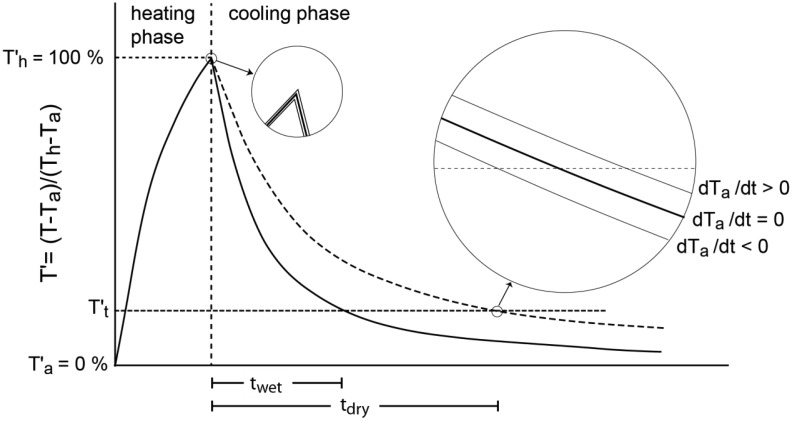
Relative Temperature *T′* = *(T* − *T_a_)*/*(T_h_* − *T_a_)* as a function of time during the measurement with a PlantCare heat dissipative sensor in a wet and a dry soil (solid and dashed lines). The effect of changes of the ambient temperature *dT_a_*/*dt* on the cooling time *t* is illustrated in the enlarged circles. (*T_a_*: ambient temperature, *T_h_*: peak temperature after heating phase at *t_o_*, *T′_t_*: threshold temperature).

The PlantCare sensor by PlantCare Ltd. (Russikon, Switzerland) consists of a heat pulse generator and a temperature sensor (SMD NTC 10 kΩ thermistors, R/T No. 8502, ±3%) embedded in a synthetic felt (Figure 1). In equilibrium with the surrounding soil, the water content of this felt is a function of the matric potential of the surrounding soil. The measurement of the PlantCare sensor consists of a heating phase and a cooling phase. In the heating phase the sensor is warmed up during 20 s with 35 mW resulting in a temperature rise of 2–3 °C, mainly depending on water content of the felt and the ambient temperature because of the strong temperature dependence of the heat conductivity. Reece [7] used this temperature rise for the calibration of the matric potential of the heat dissipation sensor. In contrast to that, the PlantCare sensor measures the time needed to cool from the peak temperature to a threshold temperature of about 20% of the temperature rise (Figure 2). The cooling time is a function of the thermal conductivity of the material around the sensor, that is mainly a function of the water content of the felt material (0.56 Wm^−1^·K^−1^ for water, 0.025 Wm^−1^·K^−1^ for air and 0.2–0.3 Wm^−1^·K^−1^ for the felt material). The large range of soil thermal conductivity of 0.3–2 Wm^−1^·K^−1^ [9,10] influences the measurement as a second order effect especially under dry conditions.

The strong temperature dependence of conventional heat dissipation sensors [8] is suppressed by the new measuring procedure with the cooling time *t* that refers to a normalized temperature change.

### 2.2. Experimental Setup

The PlantCare sensor signal was compared with measurements of other matric potential sensors as tensiometers (Soilmoisture 2710ARL06-L with additional pressure transducer). In addition to these drying experiments in pots the PlantCare sensor was also tested in an irrigated apple plantation in Conthey (Valais Canton, Switzerland) and in a tomato culture in a greenhouse.

**Figure 3.**
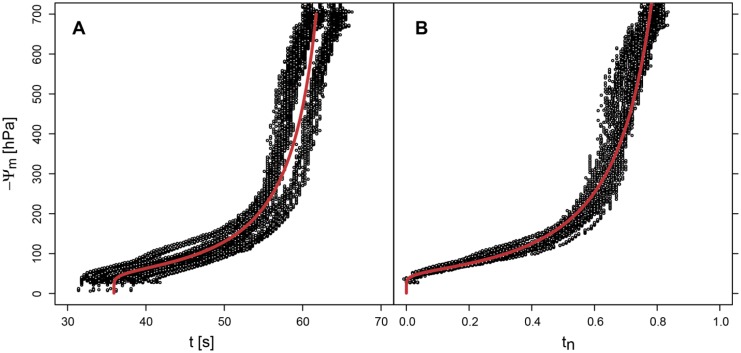
Calibration of the signals of 7 sensors with simultaneous tensiometer readings expressed as absolute cooling time *t* fitted with Equation (3) (**A**) and as normalized cooling time *t_n_* fitted with Equation (2) (**B**). The threshold temperature was 18% (Figure 2).

For the calibration of the sensor signal the sensor was put into a pressure plate apparatus (Soimoisture 1600 Pressure Plate Extractor). The calibration was done in a substrate consisting mainly of fine sand with a distribution of pore sizes similar to the synthetic felt of the sensor (see Figure 3(B) which could be considered as desorption curve of the felt).

Since the PlantCare sensors have turned out to be very sensitive to temperature changes, the experiments to establish a correction of this effect were done in a climatic chamber at constant temperature.

### 2.3. Two Point Calibration of the Sensor Signal

The differences between sensors are usually not small and there is an influence of the substrate on the measurements under dry conditions. Both problems can be minimized by normalization of the signal by two well-defined conditions: water saturated and air dried. The dimensionless cooling time *t_n_* is formulated in equation 1 as a function of the maximum cooling time *t_d_* (air dried) and the minimum cooling time *t_s_* (water saturated):

(1) $$t_{n}=\frac{t-t_{s}}{t_{d}-t_{s}}$$

Since the relation between the water content and matric potential in the porous material surrounding the sensor is similar to soils and the cooling time is a function of the water content in this porous material, the model for the water retention curve of van Genuchten [11] can be adapted to fit the normalized sensor signals:

(2) $$t_{n}=1-S=1-{\left(1+{\left|\propto \cdot \Psi_{m}\right|}^{n}\right)}^{-m}$$

The degree of saturation S is the supplement of the dimensionless cooling time *t_n_* to 1. *α, n* and *m* are fitting parameters, Ψ_m_: matric potential.

To fit the absolute cooling times *t* the water retention curve of van Genuchten [11] could be written as follows based on Equations (1) and (2):

(3) $$t=t_{s}+(t_{d}-t_{s})[1-{\left(1+{\left|\propto \cdot \Psi_{m}\right|}^{n}\right)}^{-m}]$$

where *t_s_* and *t_d_* in this formulation are additional fitting parameters to Equation (2).

### 2.4. Correction of the Influence of the Temperature Change on the Sensor Signal

The cooling time of the PlantCare sensor can be influenced by a changing ambient temperature as illustrated in Figure 2. The temperature measured in the PlantCare sensor during the heating and the cooling phase *T(t)* depends on the temperature characteristics of the sensor *T_o_(t)*, the time for heating and cooling *t_hc_* and the superimposed constant gradient of the ambient temperature *dT_a_*/*dt* (Equation (4)):

(4) $$T(t)=T_{o}(t)+t_{\text{hc}}\frac{{\text{dT}}_{a}}{\text{dT}}$$

The influence of the superimposed temperature gradient on the cooling time can be calculated for known heating and cooling curves of the sensor (see Figure 2). The corrected cooling time *t_o_* is a function of the measured cooling time *t*, the temperature gradient and the empirical factor *k*:

(5) $$t_{o}=t-k\frac{{\text{dT}}_{a}}{\text{dT}}$$

where *k* depends on the cooling time *t* and the threshold temperature $T_{t}^{\prime }$.

## 3. Results and Discussion

### 3.1. Calibration of the Sensor Signal

The pot experiments where drying was monitored by PlantCare sensors and tensiometers (Figure 3(A)) showed, that the PlantCare sensors are most sensitive between 0 and −400 hPa, that they react fast to changes in the matric potential and that the signals of the individual sensor are reproducible. But the experiments showed also, that the differences between individual sensors are substantial as it is often the case with this type of indirect matric potential sensors (e.g., [12]). In addition they showed that there is also a dependence on the substrate under dry conditions, because of the wide range of thermal conductivities of soils.

Under dry conditions the cooling time is also affected by the soil texture. An increase of the clay content by 10% lowers the cooling time by approximately 2.5%. This effect is a consequence of higher thermal conductivities of fine textured soils due to higher water contents at a similar matric potential.

The differences between the sensors and the influence of the surrounding soil are reduced using the dimensionless cooling time *t_n_* (Figure 3(B)). The difference of *t* between the air dried condition and the matric potential of −1,400 hPa is most probably the consequence of the different thermal constants of dry and moist polyamides of the felt material.

**Figure 4.**
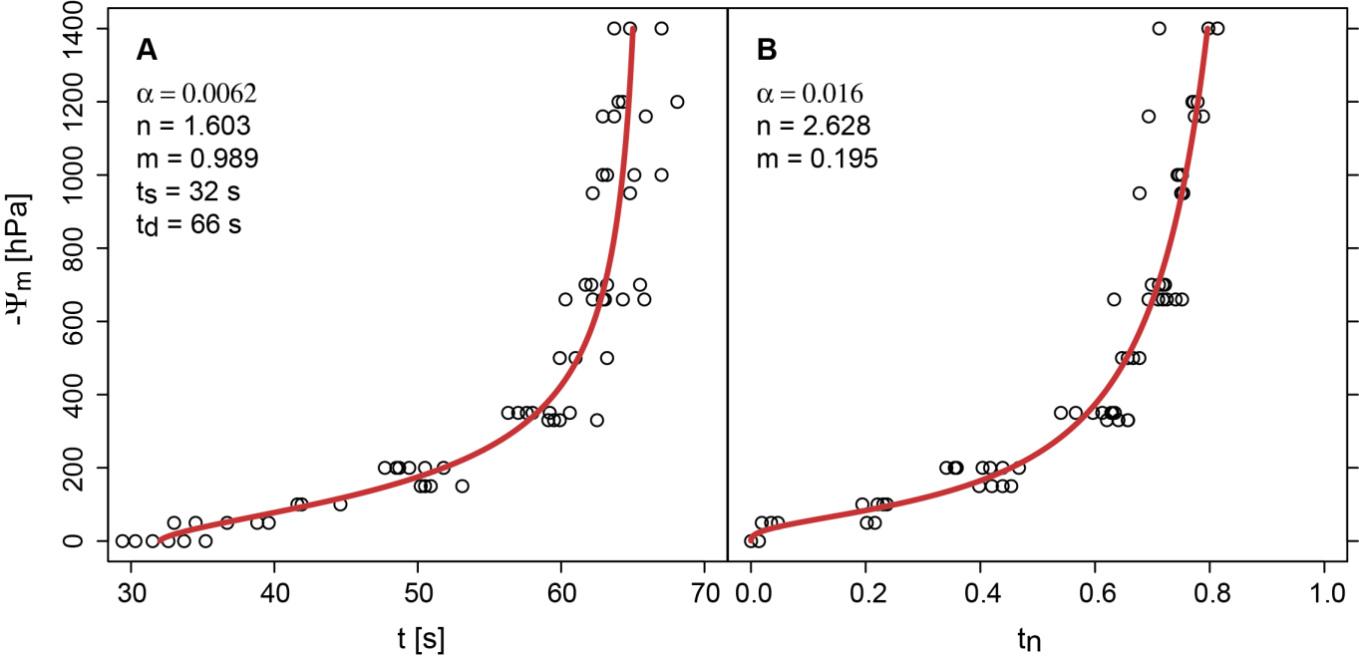
(**A**) Calibration of the PlantCare sensor signals in a pressure plate apparatus (circles) expressed as absolute cooling time *t* fitted with Equation (3) and (**B**) as normalized cooling time *t_n_* fitted with Equation (2). The threshold temperature was 18% (Figure 2).

The calibration of the sensor signal in the pressure plate apparatus (Figure 4) leads to similar results as the pot experiments with the tensiometer monitoring (Figure 3). But the pot experiments are less precise and limited by the measurement range of tensiometers at about −700 hPa.

### 3.2. Correction of the Influence of the Temperature Change on the Sensor Signal

The temperature signal of the sensor has not to be calibrated for the measurement of soil moisture because only the normalized temperature is relevant for the threshold temperature. But to explore the influence of temperature and temperature changes on the measurement of the soil moisture a temperature calibration of the sensor signal is indispensable.

The temperature signal *x* of the PlantCare sensor was calibrated with iButton temperature logger buried in the soil close to the sensor in a temperature range between 5 and 45 °C. The result of the nonlinear regression with a polynomial model is:

(6) $$T[{}^{\circ}\;C]=-5.805\cdot 10^{-9}x^{2}+1.364\cdot 10^{-3}x-13.6$$

The calibration can be approximated with an error of about 1 °C in the range of 5 to 40 °C by the following linear function:

(7) $$T[{}^{\circ}\;\text{C}]=9.58\cdot 10^{-4}x-7.1$$

**Figure 5.**
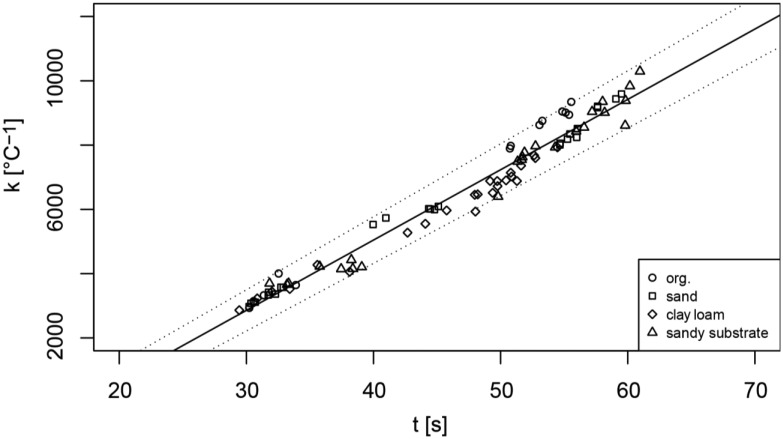
Correction factor *k* as a function of the cooling time *t* for a threshold value of 18% (Figure 2) and the 4 substrates (organic substrate, sand, clay loam and sandy substrate).

The influence of the temperature gradient on the cooling time *t* was calculated with Equation (4) for 96 heating and cooling curves. The resulting cooling times *t* leaded to correction factors *k* (Equation (5)) depending on *t* and the threshold temperature *T_t_*, where *k* increases with decreasing *T_t_* (Table 1). The linear correlation of *k* and *t* is very good and not influenced by the surrounding substrate (Figure 5).

**Figure 6.**
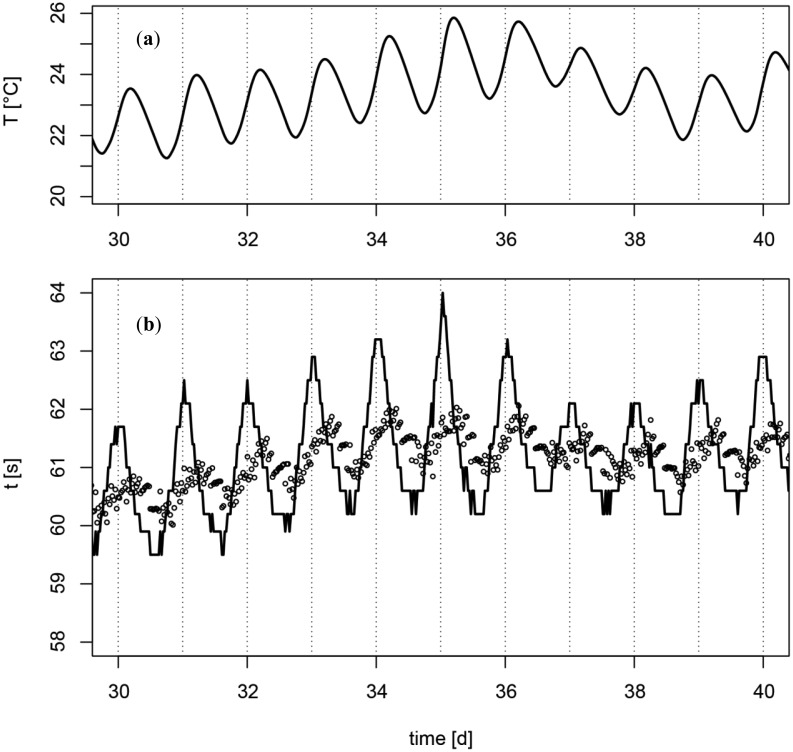
(**a**) Variations of soil temperature *T* (top) and (**b**) cooling time *t*; uncorrected (solid line) and corrected for temperature changes (circles). The sensors were placed in an irrigated apple plantation in a depth of 25 cm.

The application of the proposed correction on measurements in an irrigated apple plantation shows a remarkable reduction of the amplitude of the fluctuation (Figure 6), but the correction causes also a phase shift. Initially the oscillation of *t* was almost in phase with the oscillation of *dT_a_*/*dt* and after the correction the oscillation of *t* was in phase with the oscillation of the ambient temperature *T_a_*. The remaining fluctuation of *t* after the correction for the temperature changes could be addressed to daily variations of the matric potential due to evapotranspiration, capillary hysteresis and a weak *T* dependence ([13,14]). Another and even simpler method for correction for the influence of *T* fluctuations would be a direct adjustment of the temperature by measuring the temperature gradient immediately before each measurement cycle (Figure 2).

## 4. Conclusions/Outlook

The PlantCare sensor is a nearly maintenance-free matric potential sensor and proved appropriate to control and monitor irrigation systems. The sensor with its actual felt is most sensitive for matric potentials down to −400 hPa. Measurements between −400 and −800 hPa are possible, but imprecise. This problem could be solved with another felt characterized by finer pores.

The differences among individual sensors and the dependence of the surrounding soil type can be minimized by a two point calibration of the signal which leads to a dimensionless cooling time. If the sensor is placed close to the surface, where daily temperature fluctuations are high, a correction for the influence of the temperature changes is highly recommended.

## Figures and Tables

**Table 1.** Correction factors as a function of the cooling time *k* = *a* + *b* · *t* for values of the threshold temperature of 15%, 18% and 25%.

***T****_t_*	***a* [s^2^/°C]**	***b* [s/°C]**	**r^2^**
15%	−6,233	296	0.95
18%	−3,709	219	0.97
25%	−1,798	141	0.98
